# Causes and Mechanisms of Intrauterine Hypoxia and Its Impact on the Fetal Cardiovascular System: A Review

**DOI:** 10.1155/2010/401323

**Published:** 2010-10-19

**Authors:** Damian Hutter, John Kingdom, Edgar Jaeggi

**Affiliations:** ^1^Pediatric Critical Care Medicine and Pediatric Cardiology, University Children's Hospital, 3010 Berne, Switzerland; ^2^Department of Obstetrics & Gynecology, Mount Sinai Hospital, Toronto, ON, Canada M5G 1X5; ^3^Division of Cardiology, Hospital for Sick Children, Toronto, ON, Canada M5G 1X8

## Abstract

Until today the role of oxygen in the development of the fetus remains controversially discussed. It is still believed that lack of oxygen in utero might be responsible for some of the known congenital cardiovascular malformations. Over the last two decades detailed research has given us new insights and a better understanding of embryogenesis and fetal growth. But most importantly it has repeatedly demonstrated that oxygen only plays a minor role in the early intrauterine development. After organogenesis has taken place hypoxia becomes more important during the second and third trimester of pregnancy when fetal growth occurs. This review will briefly adress causes and mechanisms leading to intrauterine hypoxia and their impact on the fetal cardiovascular system.

## 1. Introduction

Embryogenesis, fetal growth, and survival of the perinatal period all depend on optimal maternal health and normal placental development. Maternal exposure to a persistently hypoxic environment may lead to critical injury to vital organs. Failure of the normal placental function may have profound acute and chronic effects on the developing fetus and lead to intrauterine growth restriction (IUGR), asphyxia, multiorgan failure, premature delivery, and perinatal demise. In the United States, IUGR and prematurity complicate about 12% of the deliveries and represent the leading cause of perinatal mortality and morbidity to this day, accounting for up to 75% of perinatal deaths. Long-term disabilities such as cerebral palsy, hearing loss, retinopathies, and chronic lung disease are associated with a substantial emotional burden for affected families and health care costs to the society [1]. 

In this paper, we will briefly adress relevant aspects of the normal fetomaternal physiology and then focus our attention on the causes of chronic intrauterine hypoxia and how this affects the development and performance of the fetal heart.

## 2. Normal Pregnancy

The process of placentation is initiated once the blastocyst makes contact with the epithelium of the uterus. An initial trophoblastic shell is penetrated by columns of proliferating extravillous cytotrophoblast that form the anchoring vili and provide specialized invasive cells that transform the decidual and proximal portions of the decidual spiral arteries [2]. During the initial phase of implantation and uterine wall invasion, the main role of extravillous trophoblast is to form plugs that occlude capillaries in the endometrial gland stroma; this prevents maternal hemorrhage form disrupting the conceptus and maternal blood from entering the lacunar spaces of the trophoblastic shell. Embryogenesis thus takes place in a hypoxic environment for the first 10 weeks of pregnancy because oxygen tension within the placenta is much lower than in the surrounding endometrial glands [3–6]. The “plugging” mechanism protects the growing embryo and the primitive placental villi against oxidative damage; antioxidant enzymes such as mitochondrial superoxide dismutase are not expressed by the syncytiotrophoblast before 8 to 9 weeks of gestation [7, 8]. In the period of 11–13 weeks, the trophoblastic plugs are breached by maternal blood that now enters the intervillous space. Uteroplacental blood flow increases exponentially from less than 50 mL/min in the nonpregnant state to approximately 350 mL/min by fullterm. The demands of this large rise in uteroplacental blood flow (to 20% of the total maternal cardiac output), require large adaptations in maternal physiology [9]. 

The maternal cardiac output increases by 20% to 25% during the first trimester. It reaches its peak at the beginning of the third trimester when it exceeds the prepregnancy output by 30% to 40%. This is primarily achieved by an increase in the circulating blood volume resulting in a rise in stroke volume of about 30%, by an increase in the resting heart rate of 10 to 20 beats/min and by lowering the systemic arterial blood pressure secondary to the effects of gestational hormones, circulating prostaglandins, the excessive release of human placental growth factors, and the low-resistance uteroplacental unit [10–13]. The increase of total blood volume is related to plasma expansion by 30 to 40 mL/kg body weight rather than an increase in total red blood cells and accounts for the relative anemia of pregnant women. The increased cardiac output together with the low blood viscosity lead to a rightward shift of the hemoglobin-oxygen dissociation curve [13–16]. The maternal gas exchange adapts in parallel with the hemodynamic changes. The increase in fetal-maternal oxygen demand is achieved by mild hyperventilation and anatomical changes that allow the mother to maintain her natural lung capacity despite the increase of the intra-abdominal volume. 

Increased production of endothelial nitric oxide and other vasodilators in conjunction with attenuated adrenergic vascoconstriction is thought to be responsible for maintaining uterine artery flow [17–19]. By midgestation, the human uterine artery has doubled its diameter and the increased flow is accommodated by hyperplasia of all cell layers [20–22].

### 2.1. Embryonic Heart Development

The embryonic heart develops early post conception from its origins in the heart field to a completely looped 4-chamber organ by 8 weeks of gestation [23–28]. During this period the oxygen saturation never exceeds 20%, protecting the embryo from oxidative damage [6–8]. By the time the extravillous spaces of the trophoblast are starting to be filled with maternal blood, the newly-formed fetal heart is ready to meet the increasing oxygen and nutritional demands of the growing fetus [7, 9]. The fetal oxygen saturation gradually increases during the 2nd trimester to about 60%. To maintain an adequate circulation, the fetal heart adjusts continuously to the rise in circulatory blood volume and pressure load. The right and left ventricles work in parallel, adjusting their outputs via several prenatal shunts that will close in the immediate postnatal period.

## 3. Intrauterine Hypoxia

Intrauterine hypoxia is associated with a variety of maternal, placental, and fetal conditions which may manifest differently and have different outcomes. Kingdom and Kaufmann [29] suggested to classify hypoxic pregnancy conditions into 3 subtypes: (1) *preplacental hypoxia, * where both the mother and her fetus will be hypoxic (i.e., high-altitude, cyanotic maternal heart disease; etc.); (2) *uteroplacental hypoxia*, where the maternal oxygenation is normal but the utero-placental circulation is impaired (i.e., preeclampsia, placentar insufficiency, etc.); (3) *postplacental hypoxia, *where only the fetus is hypoxic. We will focus on the first 2 subtypes as the post-placental hypoxia is mainly related to fetal diseases rather than to the direct impact of hypoxia onto the fetus.

### 3.1. Pre-Placental Hypoxia

Main causes of pre-placental hypoxia are a hypoxic environment (high-altitude) and pre-existing maternal cardiovascular disease such as cyanotic heart disease, heart failure, or pulmonary hypertension. Maternal anemia, infections, and chronic inflammation may further limit the maternal oxygen uptake and oxygen delivery to the fetus, thereby increasing the risk for adverse pregnancy outcomes. 

*Chronic hypoxia* associated with placental insufficiency plays a key role in the etiology of intrauterine growth restriction (IUGR). High-altitude exposure mimics this condition and its adverse effects on birth weight exceed those of most other risk factors for IUGR, such as maternal low weight gain, smoking, primiparity, or pre-eclampsia [30]. A 1000 meter gain in altitude results in a natural average decline of the birth weight of 100 grams [30–32]. Intrauterine growth of the chronically hypoxemic fetus generally begins to slow down between gestational week 25 to 31, a time when fetal growth normally increases exponentially [33]. Interestingly, high-altitude exposure appears also to be associated with an increased risk of pre-eclampsia that may further contribute to low birth weights in high-altitude populations [34]. Nevertheless, in most cases arterial hypertension during pregnancy at high-altitude is probably related to chronic hypoxia rather than to classic pre-eclampsia [34–36]. In line with this concept, pregnant women at high-altitude lack the physiological blood pressure fall at the beginning of the second trimester [36, 37]. A possible explanation is that chronic hypoxia diminishes the vasodilatory effect of nitric oxide while the sympathetic nervous system (*∝*_1_-/*∝*_2_-adrenergic receptor) is activated [10, 17, 18, 38–40]. In addition, potent vasoconstrictors like endothelin-1 and the hypoxia-inducible factor (HIF) are stimulated early in pregnancy by excessive generation of reactive-oxygen species (ROS) [41]. Altitude may also influence cardiac performance and the circulating blood volume. Cardiac output is lower presumably due to a lower heart rate and smaller stroke volumes related to a decreased blood volume of women living permanently at high-altitude [42, 43]. Finally, uterine arteries are typically smaller in diameter and less well perfused during pregnancy at high-altitude [44]. A direct association between uterine arterial flow and birth weight is supported by studies conducted in women from different origins [45, 46].

Women with *congenital heart disease* are at increased risk of developing pregnancy complications [47]. The probability of maternal complications has been classified as low, intermediate, or high, with estimates of 5%, 25%, and 75%, respectively, of experiencing cardiac events such as arrhythmias, pulmonary edema, stroke, or cardiac death during pregnancy [48]. The highest risk is observed in mothers with severe left-sided obstructive lesions (i.e., aortic stenosis, coarctation), pulmonary hypertension, Marfan syndrome with aortic root dilatation, as well as with symptoms of moderate or severe heart failure (NYHA functional class III and IV). Increasing maternal hypotension is the most important factor associated with intrauterine growth restriction (20% to 25%) and prematurity (20% to 25%) [49]. Interestingly, unrepaired or palliated cyanotic congenital heart disease does not belong to the high-risk group for an adverse maternal outcome but is associated with an increased risk of fetal loss. The live-birth rate is reportedly only 40% to 45% if the mother has cyanotic heart disease. This rate decreases to 10%–15% if the maternal oxygen saturation drops below 85%. In addition, extreme prematurity affects 35% to 40% of these pregnancies [50]. Fetal or neonatal death, brain hemorrhage secondary to maternal anticoagulation or to extreme prematurity, as well as IUGR are common findings in offspring of pregnant women with congenital or acquired heart disease [47, 48, 51]. 

*Chronic pulmonary disease* may have similar maternal-fetal consequences as chronic exposure to hypoxia [52]. Poorly controlled asthma is associated with pre-eclampsia, uterine hemorrhage, preterm delivery, and low birth weight [53, 54]. Among chronic lung diseases, cystic fibrosis (CF) and tuberculosis are the most common conditions: 5% of the world population is carrying the CF gene and 30% of humans have been infected with mycobacterium tuberculosis [55, 56]. Pregnancy in cystic fibrosis patients seems to have a positive effect on maternal long-term survival, despite the increased maternal risk for infections and insulin resistance and the increased fetal risk of prematurity and IUGR [57–61]. 

*Acute respiratory infections* during pregnancy are common. 1% of women experience symptoms of bronchitis or pneumonia during the course of pregnancy. Current antibiotic regimens have decreased maternal mortality from bacterial pneumonia dramatically, with the exception of cystic fibrosis. Nowadays viral pneumonias are responsible for the major part of maternal deaths during pregnancy [52]. The major risk for the fetus lies in maternal respiratory failure due to ARDS [52, 62, 63]. Fetal complications include stillbirth, spontaneous preterm labor, and a need for early delivery by Cesarean section to improve the effectiveness of maternal ventilation for respiratory failure.

*Maternal hematological disorders *may directly affect oxygen transfer. Iron deficiency anemia (IDA) is common in pregnancy and often related to malnutrition or micronutrient diets [64–67]. IDA is associated with increased risk for IUGR and prematurity [65, 68–70]. In contrast to IDA, the oxygen carrier capacity is altered in hemoglobinopathies. Sickle cell disease is particularly common in Africans and Afro-Americans [71, 72]. It may be present in combination with hemoglobin C or *β*-thalassemia (Hb S/C or Hb S/*β*). The most severe form (homozygous HbS) is called sickle cell anemia but any Hb S combination (Hb S/C or Hb S/*β*) can potentially cause vaso-occlusive crisis and hemolysis [73]. This problem is caused by the abnormal rigid sickle shape of the red blood cells with decreasing oxygen tension. Patients with sickle cell disease are at higher risk for maternal (i.e., preterm labor, preterm rupture of membranes, and postpartum infections) and fetal complications (i.e., abortion, prematurity, IUGR, low birth weight, and stillbirth) [74]. Close fetal monitoring during pregnancy and prophylactic exchange transfusion seem to be often effective in abolishing life-threatening intrauterine hypoxic events [75]. 

Thalassemia is an autosomal recessive blood disease which is particularly prevalent in Asians (*α*-form) and among Mediterranean people (*β*-form). The genetic defect results in a reduced synthesis rate of *α*- or *β*-globin chains that make up hemoglobin [73, 76]. Homozygous individuals present with severe anemia (Cooley's anemia) and extramedullary erythropoiesis. Alpha-Thalassemia major (Hb Bart's) is associated with hydrops fetalis, intrauterine death, and pre-eclampsia [71]. *β*-Thalassemia is a result of a mutation in the *β*-globin gene causing deficient or absent *β* chain production with absence of hemoglobin. The clinical picture of *β*-thalassemia varies in severity in function of the expression of Hb A. Pregnancy in thalassemia carriers is usually uncomplicated. Successful pregnancies in women with *α*- and *β*-thalassemia major have been reported but were associated with a higher incidence of IUGR, low birth weight, and prematurity [77–79].

### 3.2. Utero-Placental Hypoxia

Utero-placental hypoxia is related to abnormal placentation early in gestation and to placental vascular disease later in pregnancy. Abnormal placental implantation is a common finding in pregnancies complicated by IUGR, by gestational hypertension, and by pre-eclampsia. There exists an increased risk for both the mother and the fetus to develop cardiovascular disease later in life [68, 80–91].

**Pre-Eclampsia**

It is a complex multisystem disorder observed in human pregnancy. Maternal clinical manifestations range from mild hypertension and proteinuria to fully established HELLP syndrome (Hemolysis, Elevated Liver enzymes, Low Platelet count) or eclampsia with severe hypertension, proteinuria, and multiorgan involvement (pulmonary edema, CNS symptoms, oliguria, thrombocytopenia, and liver failure) [92–95].

Causes for its origin are largely unknown but may be the result of a systemic inflammatory response perhaps related to an immature maternal immune response [35]. Key abnormalities of pre-eclampsia include a rise in systemic vascular resistance, endothelial dysfunction, and activation of the coagulation system with enhanced platelet aggregation [92]. Endothelial dysfunction is responsible for the impaired generation and activity of vasodilators such as prostacyclin and NO and could explain surface-mediated platelet activation and fibrin formation in the uteroplacental circulation [96].

Depending on the severity of the pre-eclampsia, the condition may lead to intrauterine hypoxia and/or oxidative stress in the fetus. Pre-eclampsia is associated with IUGR and prematurity [89]. Fetal morbidity and mortality increase significantly when pre-eclampsia develops prior to 33 gestational weeks [97–100]. Pre-eclamptic mothers and their offspring are at an increased risk for premature cardiovascular disease later in life [101].

### 3.3. Post-Placental Hypoxia

In post-placental hypoxia, only the fetus becomes hypoxic which is either related to diminished uterine artery flow (i.e., mechanical compression, rupture, and thrombotic occlusion), progressive fetal cardiac failure (i.e., complete congenital heart block, complex congenital heart malformations), or due to important genetic anomalies. As mentioned earlier, we will not further explore the post-placental hypoxia as it is mainly related to fetal diseases rather than to the impact of hypoxia onto the fetus.

### 3.4. Effects of Hypoxia on the Fetus

A main consequence of chronic hypoxia is the failure of the fetus to achieve its genetically determined growth potential. About 10% of all babies grow poorly inutero and are born small for gestational age. IUGR is associated with distress and asphyxia and a 6- to 10-fold increased perinatal mortality [102]. Frequent hypoxia-mediated complications include meconium aspiration, metabolic and hematologic disturbances, cognitive dysfunction, and cerebral palsy. Acute and chronic hypoxia is also associated with a variety of morphological and functional fetal cardiac changes that aim either to compensate for the reduced oxygenation of vital organs or are the result of hypoxia-mediated fetal tissue damage [103–105].

#### 3.4.1. Hemodynamic Consequences

At an initial stage, the human fetus may be able to adapt to hypoxia by increasing the blood supply to the brain, myocardium, and upper body and decreasing the perfusion of the kidneys, gastrointestinal tract, and lower extremities. This redistribution of blood allows preferential delivery of nutrients and oxygen to the most vital organs. Cerebral vasodilatation to spare the brain from hypoxic damage leads to a decrease in left ventricular afterload while systemic arterial vasoconstriction of lower body vessels increases right ventricular afterload [106, 107]. In line with this concept, echocardiographic studies in the hypoxic fetus demonstrate an increased middle cerebral artery blood flow and a shift of the cardiac output in favor of the left ventricle [108, 109]. With further deterioration of the fetal oxygenation, this protective mechanism is overwhelmed by the decline in cardiac output and the emergence of fetal distress. The final stage is characterized by a decline in systolic and diastolic fetal cardiac function, secondary to myocardial ischemia [110]. Moreover, raised atrial contraction results in the transmission of atrial pressure waves into the venous duct and umbilical vein, causing end-diastolic umbilical vein, pulsation [111]. At this stage, reduced or reversed end-diastolic flow velocity may also be found in pulmonary veins and coronary blood flow may become visible with increased systolo-diastolic flow velocities (“heart sparing”). If not delivered, intrauterine death occurs usually within a few days [112].

In line with these findings in the hypoxic human fetus, in the hypoxic fetal sheep the cardiac output is reduced whereas the hemoglobin level is increased to maintain a near-normal oxygen delivery to the fetal myocardium [113, 114]. Moreover, in this hypoxic animal model, the coronary blood flow of the fetus is increased although there is no change in capillary/muscle fiber ratio, capillary volume density, or capillary diameter, and myocardial contractility is reduced [113–117]. 

While chronic hypoxia has detrimental consequences for the fetal heart, chronic anemia appears to have less detrimental effects because the higher oxygen affinity of fetal hemoglobin allows to compensate for this problem. In maternal anemia-related hypoxia, the fetus is able to increase the cardiac output and to increase the transplacental oxygen transfer by actively interfering with the iron metabolism of the mother. 

Surviving babies seem to be particularly susceptible to the development of arterial hypertension and cardiovascular disease later in life. An association between low birth weight and early onset of essential arterial hypertension has first been postulated by Barker in the “fetal origins of adult disease hypothesis” [118]. Barker's theory states that physiologic adaptations that enable the fetus to survive a period of intrauterine deprivation result in permanent reprogramming of the development of key organs that may have pathological consequences in postnatal life. In older children and adults, a low birth weight has been linked with increased arterial stiffness, systolic blood pressure, premature coronary heart disease, stroke and diabetes [68, 83–85, 87, 119–129], and ischemia/reperfusion injury (139–45). Despite the strong epidemiologic evidence that supports the concept of “fetal programming”, we still do not know its underlying mechanisms.

#### 3.4.2. Teratogenicity

Recently it has also been suggested that hypoxia early in gestation may be teratogenic to the human embryo. As such, maternal asthma exacerbation during the first trimester of pregnancy reportedly increased the risk for congenital malformations including the risk of cardiovascular malformations [130]. As described above, maternal blood enters the intervillous space of the human placenta only after 10 to 12 gestational weeks and until this moment the placental metabolism is anaerobic [3, 7]. Yet, the human heart forms early in the period of anaerobic metabolism between day 15 and day 60 postconception. Interestingly, if animal embryos are exposed to chronic hypoxia, cardiac malformations seem not occur more frequently.

#### 3.4.3. Cellular Effects of Hypoxia

In rats, early fetal hypoxia triggers cardiac remodeling associated with enhanced apoptosis and a significant increase in binucleated myocytes [131]. At the age of 4 months, fetal hypoxia was associated with increased heart/body weight ratio presumably due to hypertrophy of myocardium in presence of slowed fetal growth, increased *β*-/*α*-myosin heavy chain ratio, increased collagen I and III expression, and lower matrix metalloproteinase-2 activity. The consequences of these changes are higher end-diastolic pressure related to less compliant left ventricle and a reduced capability to recover from ischemia. 

Apoptosis is a controlled active physiologic process that removes unwanted or defective cells by intrinsic programmed cellsuicide [105]. In rat hearts exposed to oxidative stress, it could be shown that many genes that affect cell communication, survival and signaling were downregulated [105, 131]. This downregulation is believed to be partly responsible for the long-term consequences of intrauterine hypoxia and leaves a persistent cardiovascular “imprint” that leads to cardiovascular disease in later life. The transcription of the heat shock gen Hsp70 might be an example of this observed cardiac programming phenomenon. Hsp70 is a protein that protects against myocardial ischemia and stress (hyperthermia) and inhibits apoptosis by preventing the formation of caspase-9 [132–134]. In chronic intrauterine hypoxia conditions, the expression of Hsp70 is down-regulated [135]. This effect persists into adulthood and may explain why some adult hearts are more vulnerable against ischemia/reperfusion injury [132–138]. The expression of endothelial nitric oxide is also important for the long-term cardioprotection of the cardiomyocytes. eNOS levels are also decreased in rat hearts who were exposed to intrauterine hypoxia [139]. Similar changes were observed in the regulation of the *β*-adrenoreceptors (*β*ARs) and the coupling G proteins. *β*_2_AR and G_s_ *α* are upregulated in adult rat hearts that were inutero exposed to chronic hypoxia. This upregulation preserves cardiac contractility in hypoxia, but the regulatory mechanism appears to be lost in adulthood presumably due to wrong prenatal programming [140, 141].

## 4. Conclusion

Hypoxia does not play a major role in the early development of structural cardiac malformations probably because early embryogenesis already takes place under anaerobic conditions. Only during the second and third trimester, oxygen becomes more important for the normal fetal organogenesis and growth. If at that stage exposed to hypoxia, the fetus has a number of protective options. Immediate protection against oxidative stress is established by up-regulation of genes. Stimulation of nitric oxide synthesis enhances cell signaling for defense mechanisms, platelet inhibition, and regulation of apoptosis. *β*_2_AR and G_s_ *α* will be up-regulated to maintain a sufficient cardiac output. With persistent hypoxia, premature exit of cell cycle is initiated, together with enhanced apoptosis resulting in fewer, but hypertrophied cardiomyocytes. This process aims for better energy efficiency during hypoxic conditions but also results in less compliant ventricles. Altered regulatory gene expression in response to in-utero hypoxia appears to extend into adulthood and mimics the changes that are found in adults with chronic heart failure. Hypoxia slows fetal growth, and growth restriction is now considered a risk factor of premature arterial hypertension and cardiovascular disease, probably secondary to endothelial dysfunction. Further investigations are needed to explore preventative strategies such as the early use of antioxidants and selective vasodilators to limit the effects of intrauterine hypoxia.
